# Prognostic factors affecting long-term outcomes in patients with resected stage IIIA pN2 non-small-cell lung cancer: 5-year follow-up of a phase II study

**DOI:** 10.1038/sj.bjc.6603075

**Published:** 2006-04-04

**Authors:** D C Betticher, S-F Hsu Schmitz, M Tötsch, E Hansen, C Joss, C von Briel, R A Schmid, M Pless, J Habicht, A D Roth, A Spiliopoulos, R Stahel, W Weder, R Stupp, F Egli, M Furrer, H Honegger, M Wernli, T Cerny, H-B Ris

**Affiliations:** 1 Clinic of Medical Oncology, Hospital of Fribourg, 1700 Fribourg, Switzerland, for the Swiss Group for Clinical Cancer Research (SAKK), Bern, Switzerland

**Keywords:** chemotherapy activity, downstaging, long-term survivor, stage IIIA NSCLC

## Abstract

The aim was to investigate the efficacy of neoadjuvant docetaxel–cisplatin and identify prognostic factors for outcome in locally advanced stage IIIA (pN2 by mediastinoscopy) non-small-cell lung cancer (NSCLC) patients. In all, 75 patients (from 90 enrolled) underwent tumour resection after three 3-week cycles of docetaxel 85 mg m^−2^ (day 1) plus cisplatin 40 or 50 mg m^−2^ (days 1 and 2). Therapy was well tolerated (overall grade 3 toxicity occurred in 48% patients; no grade 4 nonhaematological toxicity was reported), with no observed late toxicities. Median overall survival (OS) and event-free survival (EFS) times were 35 and 15 months, respectively, in the 75 patients who underwent surgery; corresponding figures for all 90 patients enrolled were 28 and 12 months. At 3 years after initiating trial therapy, 27 out of 75 patients (36%) were alive and tumour free. At 5-year follow-up, 60 and 65% of patients had local relapse and distant metastases, respectively. The most common sites of distant metastases were the lung (24%) and brain (17%). Factors associated with OS, EFS and risk of local relapse and distant metastases were complete tumour resection and chemotherapy activity (clinical response, pathologic response, mediastinal downstaging). Neoadjuvant docetaxel–cisplatin was effective and tolerable in stage IIIA pN2 NSCLC, with chemotherapy contributing significantly to outcomes.

Surgery remains the best standard therapy for localised lung cancer. Results from randomised studies of early stage resectable non-small-cell lung cancer (NSCLC) have shown improved survival from use of adjuvant chemotherapy (Non-small Cell Lung Cancer Collaborative Group, 1995; Arriagada *et al*, 2004; Strauss *et al*, 2004; Winton *et al*, 2004; Douillard *et al*, 2005). Improvements in 5-year survival of 12–15% have been observed with adjuvant platinum-based doublet regimens in early stage disease (stage IB–IIIA) (Strauss *et al*, 2004; Winton *et al*, 2004; Douillard *et al*, 2005), and adjuvant chemotherapy is now considered the standard of care after radical surgical resection. However, the associated toxicity and subjective poor tolerance of adjuvant chemotherapy are a major limitation. Patient and physician compliance is low and even in trials only 35–85% of targeted dose intensity has been achieved. Moreover, a large randomised intergroup trial failed to demonstrate any survival advantage when cisplatin and etoposide were added to postoperative radiation, compared with radiation alone for resected stage II–IIIA disease (Keller *et al*, 2000).

Several small randomised studies (Pass *et al*, 1992; Rosell *et al*, 1994; Roth *et al*, 1994) have demonstrated a benefit from neoadjuvant treatment in locally advanced disease (stage IIIA). In a large trial involving 355 patients with stage IB–IIIA NSCLC, a trend towards survival benefit was seen from neoadjuvant chemotherapy (median survival 37 months *vs* 26 months for surgery alone; *P*=0.15) (Depierre *et al*, 2002). Encouragingly, preoperative chemotherapy appears to be better tolerated than adjuvant chemotherapy, with a high compliance of 90–95% (Depierre *et al*, 2002; Betticher *et al*, 2003). Similarly, preliminary results of a recent randomised trial of preoperative chemotherapy (paclitaxel and carboplatin) have shown a nonsignificant trend towards improved progression-free survival and overall survival (OS) (Pisters *et al*, 2005).

The routine use of neoadjuvant chemotherapy in NSCLC remains controversial. Identification of predictive/prognostic factors to select patients who would derive the greatest benefit from combined modality therapy will help clarify the most appropriate clinical use of neoadjuvant chemotherapy, particularly for patients with stage IIIA disease. We report here the final and updated results of a large phase II trial in patients with pN2 (proven by mediastinoscopy) stage IIIA (International Union Against Cancer, UICC) disease. Analyses of potential factors associated with the development of local relapse and distant metastases are also presented.

## PATIENTS AND METHODS

### Patient characteristics

Initial results of this prospective, multicentre, phase II trial of docetaxel–cisplatin induction chemotherapy have been reported previously (Betticher *et al*, 2003). Briefly, all patients were required to have mediastinoscopically proven, previously untreated, operable stage IIIA (T1–3 pN2 M0) NSCLC. Eligibility criteria included: age 18–75 years; World Health Organization (WHO) performance status (PS) ⩽2; forced expiratory volume ⩾1.2 l s^−1^; normal cardiac and bone marrow (leucocyte >4.0 × 10^9^ l^−1^, platelet >100 × 10^9^ l^−1^) functions and adequate hepatic (bilirubin within normal limits, aspartate aminotransferase and alanine aminotranferase ⩽1.5 × upper limit of normal (ULN), alkaline phosphatase ⩽2.5 × ULN) and kidney (creatinine clearance >60 ml min^−1^) functions. The trial was approved by the local ethics committee at each participating centre, with informed, written consent obtained from all patients.

### Combined modality therapy

Patients were scheduled to receive cisplatin 80 mg m^−2^ as a 1-h infusion (divided over 2 days, on days 1 and 2) plus docetaxel 85 mg m^−2^ as a 1-h infusion on day 1, every 3 weeks for three cycles providing haematological function was adequate (neutrophil >1.5 × 10^9^ l^−1^, platelet >100 × 10^9^ l^−1^). In view of the low toxicity observed in the first 36 patients, the protocol was subsequently amended to increase the dose of cisplatin to 100 mg m^−2^ (50 mg m^−2^ on days 1 and 2) from patient 37 onwards. Resection was performed if there was no progressive disease (PD) based on post-induction computed tomography (CT) scan and no contraindication emerging from pulmonary function testing. Postoperative mediastinal radiotherapy was to be administered to patients with positive resection margin (R1 and R2) and/or involvement of the uppermost mediastinal lymph node. A daily dose of 2 Gy delivered in the central axis at the mid-plane was administered 5 days/week to a cumulative dose of 60 Gy. The irradiated field included the bronchial stump, ipsilateral hilum and vascular shadows of the mediastinum bilaterally. Postoperative chemotherapy was not administered.

### Evaluations

One of the trial objectives was to identify possible prognostic factors of the combined modality treatment (chemotherapy, surgery and optional radiotherapy). We assessed the association of OS, event-free survival (EFS; an event comprising PD, relapse or death), risk of local relapse and risk of distant metastases with each of 19 potential prognostic factors: baseline patient characteristics (age, gender, PS, smoking habits, serum lactate dehydrogenase (LDH) and haemoglobin); baseline tumour characteristics (histology, tumour stage, differentiation, multilevel involvement of mediastinal lymph nodes, and nodal enlargement on CT scan); type of surgery performed (right pneumonectomy, resection margin and complete resection) and activity of chemotherapy (clinical response, pathological responses (⩾95% and <60%/⩾60%; this cutoff represented the median amount of necrosis and fibrosis of all tumours), mediastinal downstaging, involvement of the uppermost mediastinal lymph node). Response to chemotherapy was evaluated using WHO criteria and a complete pathological response was defined as ⩾95% necrosis and fibrosis. Each patient's CT scan was assessed by the respective centre's medical panel and was reviewed centrally based on the local reports. After surgery/radiotherapy, patients were seen every 3 months and investigated by CT scan until recurrence or death. The rate of relapse (local relapse or distant metastases) was recorded.

Histological diagnosis and assessment of mediastinal lymph nodes were performed using American Thoracic Society mapping criteria (Mountain and Dresler, 1997) in all patients. Histology was reviewed centrally by an experienced pneumopathologist; any discrepancies were discussed with local pathologists in order to establish a consensus on the diagnosis. Results of CT scans, mediastinoscopy and surgical procedures were also reviewed centrally, based on reports provided.

### Statistical analysis

Only patients who underwent surgery after induction chemotherapy were included in the present analysis. Continuous variables were analysed by descriptive statistics and categorical variables by frequency tables. Overall survival was determined from the time of enrolment to death, while EFS was calculated as the interval between enrolment and PD, relapse, or death. Cure was defined as no relapse within 3 years. In some analyses, time to death due to tumour was calculated as OS; however, with deaths not due to tumour, patients were censored at their dates of death. Time to local relapse was similarly defined, ending at the date of documented local relapse or death due to tumour. Time to distant relapse was defined as the time between enrolment and documented metastases or tumour-related death. Time-to-event variables (e.g. OS, EFS) were estimated using the Kaplan–Meier method. Comparisons between groups were performed using the Wilcoxon rank-sum test for continuous variables, the χ^2^ or Fisher's exact test for categorical variables and the log-rank test for time-to-event variables.

The prognostic impact of certain variables on time-to-event outcomes was investigated using the Cox proportional hazards model. The association between an outcome and a potential prognostic factor was investigated separately for each individual factor (univariate analysis). The prognostic impact of mediastinal downstaging (N0–1 *vs* N2), complete resection and the activity of chemotherapy on the primary tumour was also investigated in multivariate analyses including four additional established factors (age, PS, tumour stage and LDH). All *P*-values are two-sided. No correction was performed for multiple evaluations.

## RESULTS

### Patients and treatments

In total, 90 patients with stage IIIA pN2 NSCLC were enrolled and were assessable for toxicity and response following docetaxel–cisplatin chemotherapy (Betticher *et al*, 2003). Chemotherapy was well tolerated and active, with 95% of the planned dose and cycles administered, and an overall response rate of 66% (including complete remissions in 8% and partial remissions in 58% of patients; PD was seen in 10% of patients) (Betticher *et al*, 2003).

In all, 75 patients underwent tumour resection after three cycles of chemotherapy, with positive resection margin in 16% of patients (Betticher *et al*, 2003). Perioperative morbidity and mortality were low (17 and 3%, respectively). Complete pathological response was seen in 19% of resected patients. In all, 33 patients (44%) received radiotherapy, with median total dose 60 Gy (range 22–70). Of these, 23 patients were treated per protocol (i.e. for a positive margin and/or involvement of the upper lymph node). Nine patients who were due to receive postoperative radiotherapy did not actually receive this treatment. Owing to the small numbers of patients involved, statistical comparison between these patient groups was considered unfeasible. Table 1 summarises the characteristics of the 75 patients who underwent surgery, including the types of resection and response to chemotherapy. All results presented hereafter relate to this subset of 75 patients unless otherwise stated.

### Overall survival and EFS

**Figure 1:**
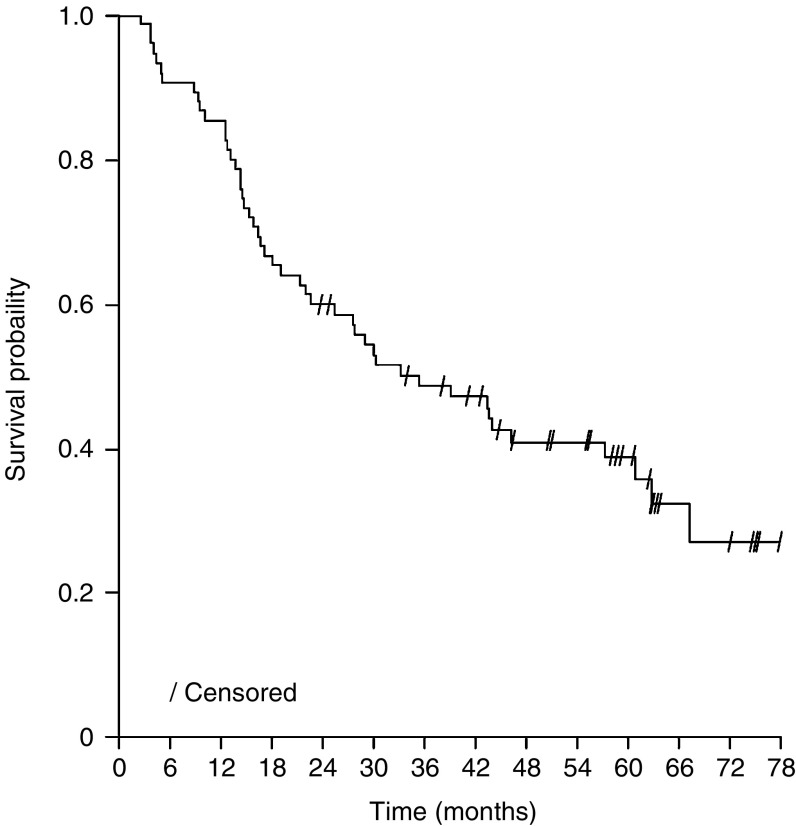
Overall survival (patients who underwent surgery, *n*=75).

After a median observation time of 5 years, the median OS was 35 months (Figure 1) and the median EFS was 15 months; corresponding survival figures for all 90 patients enrolled were 28 months and 12 months, respectively. Of 47 patients who died, 42 were due to tumour. The median time to death due to tumour was 43 months. At 3 years after initiation of trial therapy (a follow-up time which all patients reached), 27 patients (36%) were alive and tumour free (cured). Late toxicities (dyspnoea, chest pain, myelotoxicity) due to the combined modality treatment were not seen.

### Prognostic factors for survival and cure

Univariate analysis (Table 2) assessing the impact of baseline patient and tumour characteristics identified only multilevel involvement of mediastinal lymph nodes as a poor prognostic factor for OS. Among surgery characteristics, only complete resection was significantly associated with better OS and EFS. All factors relating to activity of the chemotherapy were associated with better OS and EFS. Similar associations were seen when patients who died from nontumour causes were censored (data not shown). The prognostic impacts of complete tumour resection, mediastinal downstaging and pathological response on OS and EFS were confirmed in multivariate analyses (data not shown).

In a further univariate analysis, prognostic factors for cure were identified as: clearance of the uppermost mediastinal lymph node after chemotherapy (23 out of 42 cures in patients with clearance *vs* two out of 25 cures in patients without clearance; *P*=0.0002); ⩾60% necrosis and fibrosis in the primary tumour after chemotherapy (19 out of 37 *vs* seven out of 33; *P*=0.01); mediastinal downstaging (20 out of 43 *vs* four out of 25; *P*=0.02) and complete resection (23 out of 41 *vs* four out of 31; *P*=0.0002). Smoking habits (current or former), PS at start of therapy and age had no influence on disease evolution.

### Localisation of relapses

As the first event, local relapse and distant metastases occurred in 14 out of 75 (19%) and 26 out of 75 (35%) patients, respectively. A further eight out of 75 (11%) patients had both local relapse and distant metastases. At longer follow-up, two out of 75 (3%) patients had local relapse only, six out of 75 (8%) distant relapse only, 43 out of 75 (57%) had both local and distant relapse, and 24 out of 75 (32%) patients did not develop either relapse. In univariate analysis, the risk of local relapse and of distant metastases, including death due to tumour in both cases, was significantly associated (in terms of lower risk) with complete tumour resection and chemotherapy activity (CT-assessed clinical response, pathological response, mediastinal downstaging and clearance of the uppermost mediastinal lymph node) (Table 2). Furthermore, there were trends toward increased risk of distant metastases (*P*=0.04) and local relapse (*P*=0.07) in patients with a positive resection margin. The prognostic impacts of complete tumour resection, pathological response to chemotherapy and mediastinal downstaging on the risks of developing local and distant relapses were confirmed in multivariate analyses (Table 3).

The distribution of distant relapses is shown in Table 4. Interestingly, recurrence occurred most frequently in the lung (other lobe and/or opposite lung). In all, 10 patients (13%) developed brain metastases as the first relapse and three (4%) at later relapses. Of the patients, 10 developed brain metastases within 12 months of enrolment. Females (five out of 17 *vs* five out of 58 males; *P*=0.04) and patients with a positive resection margin (four out of 12 *vs* six out of 63 with a negative resection margin; *P*=0.05) were at a higher risk of developing brain metastases within the first 12 months.

### Associations of chemotherapy activity and outcomes

As shown in Table 2, all aspects of chemotherapy activity (clinical response, pathological response, mediastinal downstaging and clearance of the uppermost mediastinal lymph node) were significantly associated with improved OS and EFS. Pathological response (percentage of necrosis and fibrosis) was the most important feature of the chemotherapy activity on the primary tumour. The median value of pathological response was 60%; this was taken as the main cutoff point to dichotomise the variable, although calculations with other cutoff points were also performed. In our study, preoperative CT scan was a relatively poor way to restage patients after neoadjuvant chemotherapy and often underestimated the degree of local tumour regression. Indeed, 20 patients with stable disease had a median of 47.5% (range 0–100%) necrosis and fibrosis and in three patients a complete (⩾95%) pathological response was found. Four patients with stable disease at resection and without any recurrence within 3 years had a median of 75% (30–100%) necrosis and fibrosis. Tumours of 26 patients who were relapse-free within 3 years of enrolment showed a median of 85% (0–100%) necrosis and fibrosis.

**Figure 2:**
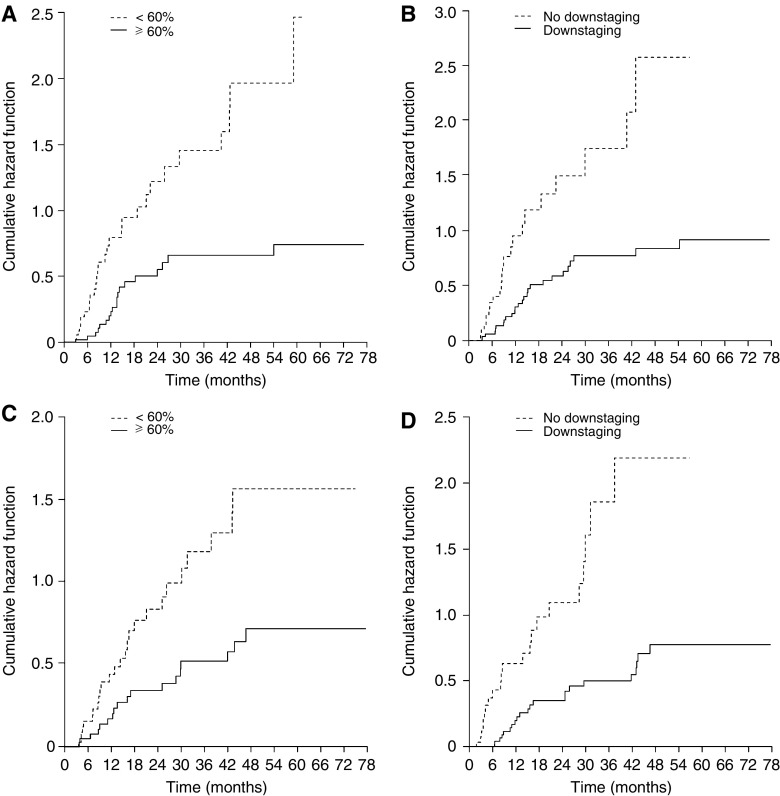
Risk of (**A**) distant metastases stratified by pathological response (percentage of tumour necrosis and fibrosis), (**B**) distant metastases stratified by mediastinal downstaging (N0/1 *vs* N2), (**C**) local relapse stratified by pathological response and (**D**) local relapse stratified by mediastinal downstaging. The risk of developing distant metastases decreased rapidly after 24 months in patients where chemotherapy was active. Conversely, local relapses occurred throughout the entire observation period.

The risk of developing distant metastases was almost zero after 27 months if the chemotherapy induced ⩾60% necrosis/fibrosis in the tumour (Figure 2A). Conversely, local relapse was seen until 4 years after enrolment, although chemotherapy was active as shown by the amount of necrosis and fibrosis in the tumour and mediastinal downstaging (Figure 2B and D). Importantly, for risk of distant metastases and of local relapse, the hazard ratios of the group with high pathological response *vs* the group with low response decreased as the cutoff point increased (*P*⩽0.01). When comparing patients who achieved a complete pathological response (⩾95% necrosis and fibrosis, the largest cutoff point; 13 (16%) patients) with those who did not, the hazard ratios for local relapse and for distant metastases were 0.21 and 0.26, respectively.

## DISCUSSION

The final analysis of the study data after a longer follow-up (median 5 years) confirms and strengthens our previous conclusions (Betticher *et al*, 2003). The median survival of the 90 patients included in the trial and for the 75 resected patients was 28 months and 35 months, respectively. To our knowledge, these are among the best results obtained in stage IIIA pN2 NSCLC patients. In a similar group of patients who had received three cycles of cisplatin and gemcitabine preoperatively, the median survival was 18.9 months (Van Zandwijk *et al*, 2000), while two other trials reported median survival times of 19 and 22 months for patients with stage IIIA pN2 NSCLC who received neoadjuvant chemotherapy (Martini *et al*, 1993; Rosell *et al*, 1999).

We sought to identify characteristics of the neoadjuvant chemotherapy, as well as other prognostic factors, which would allow enhancement of therapy and help select patients for thoracotomy. Several factors have been previously described that predict favourable long-term outcome from a multimodality approach, including clinical and pathological response to chemotherapy, the ability to completely resect the tumour and the mediastinal lymph nodes, and complete clearance of N2 disease (Albain *et al*, 1995; Elias *et al*, 1997; Burkes *et al*, 2005). In agreement with these results, we found that chemotherapy activity at the primary tumour, mediastinal downstaging and complete tumour resection (with a negative uppermost mediastinal lymph node) were strongly associated with improved OS and EFS, and reduced risk of local and distant relapse. Our identification of nodal downstaging from N2 to N1 and/or N0 as a powerful prognostic marker is consistent with findings by Albain *et al* (1995), who found that mediastinal nodal downstaging (to N0) was predictive of improved survival in IIIA/IIIB patients, although inclusion of preoperative radiotherapy makes it difficult to evaluate the independent effect of neoadjuvant chemotherapy on this outcome. Similarly, in a retrospective analysis of 103 patients who underwent neoadjuvant therapy and resection for stage IIIA pN2 disease, downstaging to N0 was associated with improved 5-year survival compared with patients who were N2 or N1 after chemotherapy (no difference in survival was seen between N2 and N1 patients in this analysis) (Bueno *et al*, 2000).

We demonstrated that the activity of the chemotherapy on the tumour and on lymph nodes, as characterised by the amount of necrosis and fibrosis and clearance of the malignant cells, respectively, was highly associated with outcomes. Several trials assessing the role of neoadjuvant chemotherapy, as compared to local treatment (surgery or radiotherapy), demonstrated a reduction of the risk of distant metastases (Le Chevalier *et al*, 1991; Sause *et al*, 1995; Dillman *et al*, 1996; Depierre *et al*, 2002). To our knowledge, our data show for the first time that the risk of local relapse can be reduced in relation to the extent of chemotherapy activity on the primary tumour. In fact, in patients with ⩾60% pathological response, the risk of local relapse and distant metastases was reduced by 56 and 64%, respectively. In patients with a lesser response, local relapse was frequent (60% of patients) and occurred up to 4 years after enrolment. Conversely, most cases of distant metastases were seen within 2 years following enrolment, after which the risk decreased almost to zero. Therefore, our efforts should also be concentrated on further reducing the risk of local relapse alongside reduction of distant metastases.

In our study, the most frequent site of distant metastases was the lung. This contrasts with the results reported by others who described the brain as the most common site of recurrence (Burkes *et al*, 1992, 2005; Albain *et al*, 1995; Darwish *et al*, 1995; Choi *et al*, 1997; Eberhardt *et al*, 1998; Mamon *et al*, 2005). Approximately, 40% of patients with stage IIIA NSCLC treated with preoperative chemotherapy and surgical resection have been reported to develop brain metastases (Andre *et al*, 2001; Mamon *et al*, 2005). The risk of developing brain metastases appears to be associated with nonsquamous histology (Robnett *et al*, 2001; Mamon *et al*, 2005) and with the type of chemotherapy. Indeed, in one retrospective analysis, treatment with a taxane – platinum-containing regimen was associated with a lower risk of brain metastases than other platinum-based combinations (25 *vs* 52%, respectively) (Mamon *et al*, 2005). Unexpectedly, in our study we found a lower incidence of brain metastases: 13% as first relapse site and 3% at later relapse (17% in total); 10 of 13 relapses occurred within the first 12 months. Female subjects seemed to be at a higher risk of developing brain metastases. The low number of cases, however, does not allow further conclusions. The role of prophylactic cranial irradiation needs to be carefully investigated and weighed against late toxicity in patients receiving neoadjuvant docetaxel and cisplatin, as this regimen apparently may reduce the risk of the development of brain metastases.

Analysis of resected tissue after chemotherapy identified a group of patients already destined for long-term survival due to tumour eradication by induction therapy. In these cases, surgical resection perhaps acted more as a diagnostic and prognostic tool to select patients for complete resection rather than providing therapeutic benefit. However, the persistence of malignant cells in the uppermost mediastinal lymph node following chemotherapy was associated with a higher risk of local relapse and development of distant metastases. Moreover, patients without any relapse within 3 years had tumours with a median of 15% viable cells. These findings suggest that further therapy is crucial for long-term survival, even in patients who respond to induction chemotherapy; however, it is not clear whether surgery is the best means to achieve this. Results from a phase III trial comparing chemotherapy and radiotherapy with preoperative chemotherapy and surgical resection in patients with stage IIIA pN2 NSCLC found no survival differences between treatment arms (Johnstone *et al*, 2002). In contrast, the final results from a study conducted by the North American Intergroup, in which patients with stage IIIA NSCLC were randomised to postinduction resection or further chemotherapy and radiotherapy following induction with chemoradiotherapy, showed improved progression-free survival in the surgery arm (Albain *et al*, 2003, 2005). The European Organisation for Research and Treatment of Cancer (EORTC) study (INT 08941) randomised patients with nonresectable locally advanced stage IIIA disease to cisplatin-based induction chemotherapy followed either by surgery or radiotherapy. Survival was comparable with either strategy (van Meerbeeck *et al*, 2005).

The lack of consistent restaging after induction chemotherapy, and the difficulty in accurately assessing tumour response shortly after completing the induction treatment, limits the comparability between studies and strategies. In several studies, and most notably in the recently reported EORTC trial, patients were excluded from attempted surgical resection in the absence of a response to induction therapy. In fact, patients with ‘clinically stable disease’ following neoadjuvant therapy not uncommonly have major pathological response at the time of surgery, as has been observed previously (Albain *et al*, 1995) and in the present trial. Clearly, better methods of assessing response after preoperative therapy are needed. Positron emission tomography may be a more accurate tool for assessing tumour response and is currently being prospectively investigated in several trials.

In conclusion, our results with neoadjuvant docetaxel and cisplatin in patients with locally advanced NSCLC (stage IIIA pN2) who underwent surgical resection revealed that, after a median follow-up of 5 years, a cure rate of 36% of resected patients can be obtained; also, the activity of chemotherapy on the primary tumour and on mediastinal lymph nodes correlate with improved OS and EFS, and reduced risk of local relapse and distant metastases. In addition, we observed a high rate of local relapse, thereby encouraging the further investigation of radiotherapy in this patient group. Finally, the risk of brain metastases was lower than that reported in previous trials.

## Figures and Tables

**Table 1:** Characteristics of patients (*n*=75), surgery and chemotherapy

**Characteristic**	**No. of patients**	**%**
*Patient characteristics*		

Gender		
Female	17	23
Male	58	77
		
Age (years); median (range)	59 (39–76)	
		
Performance status		
0	45	60
1	28	37
2	2	3
		
Number of pack-years; median (range)	45 (9–160)	
		
Smoking habits		
Continued	31	41
Stopped	37	49
Never smoked	7	10
		
Serum LDH (× ULN); median (range)	0.77 (0.54–3.56)	
>1 × ULN	14	19
		
*Tumour characteristics*		
		
Initial histology (pathologist from participating centres)		
Squamous cell carcinoma	32	43
Adenocarcinoma	23	31
Large cell carcinoma	9	12
Poorly differentiated NSCLC	11	14
		
Tumour stage		
1	6	8
2	44	59
3	25	33
		
Mediastinal lymph node enlargement (>1 cm) by CT scan		
Yes	61	81
No	14	19
		
Tumour localisation		
Upper lobe right	31	42
Middle lobe right	3	4
Lower lobe right	9	12
Upper lobe left	11	15
Lower lobe left	1	1
Central right	10	13
Central left	10	13
		
*Characteristics of the surgery*		
Type of resection:		
Lobectomy/bilobectomy	28/10	37/13
Pneumonectomy (right/left)	21/16	28/22
Complete resection^a^		
Yes	43	57
No	32	43
Radical resection of the primary tumour:		
Negative margin	63	84
Positive margin	12	16
		
*Activity of the chemotherapy*		
		
Response after neoadjuvant chemotherapy		
CR	7	9
PR	48	64
NC	20	27
		
Pathological response on primary tumour (% of necrosis and fibrosis, two missing)		
Median (range)	60% (0–100%)	
⩾90%	20	27
<20%	12	16
		
Downstaging of mediastinal lymph nodes (four missing)		
N0	23	32
N1	22	31
N2	26	37

CT=computed tomography; LDH=lactate dehydrogenase; NSCLC=non-small-cell lung cancer; ULN=upper limit of normal.

a Complete resection: negative resection margin (R0) and no involvement of the uppermost mediastinal lymph node in the mediastinal lymphadenectomy preparation.

**Table 2:** Prognostic factors for overall survival, event-free survival and disease relapse (distant or local): univariate analysis

		**Overall survival (months)**		**Event-free survival (months)**		**Local relapse (months)**		**Distant metastases (months)**	
**Factor**	**Characteristics**	**Median**	***P***	**Median**	***P***	**Median**	***P***	**Median**	***P***
*Baseline patient characteristics*									
	Overall (months)	35.1	—	14.8	—	29.9	—	18.9	—
1	Age (⩽59/⩾60 years)	35.1/30.0	0.40	11.6/15.5	0.66	25.2/30.3	0.53	13.0/24.6	0.45
2	Gender (female/male)	46.1/27.4	0.15	30.3/12.5	0.34	43.3/25.2	0.33	30.3/15.5	0.73
3	PS (0/1–2)	39.0/34.0	0.54	19.5/13.6	0.95	30.3/29.9	0.44	25.9/14.8	0.72
4	Smoking (pack-years ⩽45/>45)	39.0/27.4	0.48	14.9/14.8	0.67	31.7/21.2	0.32	21.8/15.5	0.87
	Smoking (continued/stopped or never)	29.9/39.0	0.96	12.3/21.1	0.56	16.6/42.1	0.22	14.2/24.6	0.45
5	LDH (⩽1/>1 × ULN)	35.1/21.6	0.49	12.3/13.6	0.49	26.3/25.2	0.46	21.8/14.5	0.72
6	Haemoglobin (⩽/> 134 g l^−1^ (median)	29.9/43.8	0.38	13.6/25.2	0.29	26.3/43.4	0.17	15.4/27.4	0.34
									
*Baseline tumour characteristics*									
7	Histology (squamous/adenocarcinoma+other)	30.0/39.0	0.75	19.2/12.7	0.10	31.7/26.3	0.55	22.8/15.4	0.08
8	Tumour stage (T1–2/T3)	27.6/57.1	0.12	13.7/15.5	0.12	26.3/NR	0.14	21.8/15.5	0.40
9	Differentiation (G1–2/G3)	30.0/46.1	0.69	14.7/14.0	0.58	29.9/26.3	0.98	15.5/18.9	0.73
10	N multilevel (no/yes)	43.4/21.8	0.05	15.5/9.8	0.47	31.7/12.6	0.36	19.5/12.6	0.39
11	N enlargement on CT scan before chemotherapy (⩽1 cm/>1 cm)	32.5/29.9	0.47	8.9/12.7	0.71	18.9/26.3	0.72	9.3/15.4	0.50
									
*Characteristics of the surgery*									
12	Right pneumonectomy (no/yes)	30.0/46.1	0.56	13.6/26.3	0.36	29.9/26.3	0.58	15.6/40.9	0.27
13	Resection margin of primary tumour (negative/positive)	43.3/16.5	0.10	15.5/11.4	0.11	31.7/15.8	0.07	22.8/12.4	0.04
14	Complete tumour resection^a^ (yes/no)	62.5/17.3	<0.0001	42.1/9.6	<0.0001	NR/14.4	<0.0001	54.5/13.0	<0.0001
									
*Activity of the chemotherapy*									
15	Clinical response (CR+PR/NC)	43.8/20.0	0.03	22.8/9.2	0.02	43.3/16.2	0.004	24.6/9.4	0.03
16	Complete pathological response (⩾95%) (no/yes)	28.8/NR	0.04	12.4/62.5	0.008	25.2/NR	0.005	15.4/NR	0.006
17	Pathological response (<60%/⩾60%)	22.4/60.6	0.03	9.0/42.1	<0.0001	16.6/46.9	0.0009	11.7/54.5	<0.0001
18	Mediastinal downstaging (N0/N1/N2)	NR/35.1/16.4	0.0001	42.1/15.8/8.6	0.0003	NR/25.2/14.4	<0.0001	NR/19.5/9.3	0.0003
	Mediastinal downstaging (N0/N1+N2)	NR/22.1	0.001	42.1/11.4	0.008	NR/17.1	0.001	NR/14.2	0.005
	Mediastinal downstaging (N0+N1/N2)	57.1/16.4	<0.0001	25.2/8.6	<0.0001	43.8/14.4	<0.0001	26.4/9.3	0.0001
19	Uppermost mediastinal lymph node involved (no/yes)	62.5/17.3	<0.0001	39.4/9.2	<0.0001	46.9/14.4	<0.0001	43.2/12.2	<0.0001

CR=complete response; CT=computed tomography; LDH=lactate dehydrogenase; N=node; NC=no change; NR=median value not reached; *P*=log-rank test *P*-value; PD=progressive disease; PR=partial response; PS=performance status; ULN=upper limit of normal.

a Complete tumour resection: negative resection margin and no involvement of the uppermost mediastinal lymph node.

**Table 3:** Multivariate analyses for risk of developing local or distant relapse

	**Univariate**				**Multivariate model 1**			**Multivariate model 2**			**Multivariate model 3**		
**Characteristic**	***N***	**Hazard ratio**	**95% CI**	***P***	**Hazard ratio**	**95% CI**	***P***	**Hazard ratio**	**95% CI**	***P***	**Hazard ratio**	**95% CI**	***P***
*(a) Prognostic factors for time to local relapse or death due to tumour in univariate and multivariate analyses*													
Age ⩾60	75	0.83	0.46–1.49	0.53	0.55	0.29–1.05	0.07	0.65	0.35–1.22	0.18	0.771	0.399–1.491	0.44
PS 1–2	75	0.79	0.43–1.45	0.44	0.78	0.37–1.63	0.51	0.95	0.46–1.95	0.89	0.872	0.424–1.794	0.71
Tumour stage 3	75	0.61	0.31–1.19	0.14	0.76	0.35–1.65	0.49	0.49	0.23–1.05	0.06	0.480	0.220–1.047	0.07
LDH >1 × ULN	66	1.31	0.64–2.68	0.46	1.23	0.58–2.64	0.59	1.09	0.52–2.29	0.83	1.243	0.590–2.621	0.57
Mediastinal downstaging	71	0.30	0.16–0.55	0.0001	0.29	0.15–0.59	0.0006	—	—	—	—	—	—
Complete tumour resection	75	0.28	0.15–0.52	<0.0001	—	—	—	0.28	0.14–0.57	0.0003	—	—	—
Pathological response ⩾60%	73	0.444	0.242–0.813	0.0085	—	—	—	—	—	—	0.523	0.263–1.038	0.06
													
*(b) Prognostic factors for time to distant relapse or death due to tumour in univariate and multivariate analyses*													
Age ⩾60	75	0.81	0.46–1.41	0.45	0.58	0.32–1.07	0.08	0.72	0.40–1.29	0.27	0.789	0.425–1.461	0.45
PS 1–2	75	1.11	0.63–1.97	0.72	1.08	0.54–2.15	0.84	1.33	0.68–2.59	0.41	1.454	0.727–2.909	0.29
Tumour stage 3	75	0.77	0.41–1.43	0.40	0.94	0.44–1.99	0.87	0.60	0.30–1.22	0.16	0.589	0.280–1.239	0.16
LDH >1 × ULN	66	1.14	0.56–2.31	0.72	1.15	0.53–2.51	0.72	1.07	0.51–2.24	0.85	1.099	0.521–2.320	0.80
Mediastinal downstaging	71	0.34	0.19–0.60	0.0003	0.32	0.16–0.62	0.0008	—	—	—	—	—	—
Complete tumour resection	75	0.32	0.17–0.58	0.0002	—	—	—	0.33	0.18–0.64	0.0009	—	—	—
Pathological response ⩾60%	73	0.356	0.199–0.639	0.0005	—	—	—	—	—	—	0.347	0.181–0.663	0.001

CI=confidence interval; LDH=lactate dehydrogenase; *P*=Wald test *P*-value; PS=performance status; ULN=upper limit of normal.

CI=confidence interval; LDH=lactate dehydrogenase; *P*=Wald test *P*-value; PS=performance status; ULN=upper limit of normal.

Although mediastinal downstaging, complete tumour resection and pathological response (⩾60%) were associated with overall survival in the univariate analyses, they also correlated with each other. It was not possible to include all of these factors in the multivariate model (this table presents the updated analysis of our previous report (Betticher *et al*, 2003)).

**Table 4:** Sites of distant metastases (at time of first diagnosis and at longer follow-up) in 75 patients. The site of metastases was documented in 37 of 49 patients, with 1–3 sites per patient (total of 54 sites)

**Site**	**Number of patients**	**% of patients/sites**	**95% confidence interval for patients (%)**
Lung and pleura	18	24/33	15–35
Brain	13	17/24	10–28
Lymph nodes	7	9/13	4–18
Liver	6	8/11	3–17
Skeleton	4	5/7	1–13
Skin	3	4/6	0.8–11
Adrenal glands	2	3/4	0.3–9
Peritoneal carcinoma	1	1/2	0.03–7
